# Substitution

**Published:** 1898-02

**Authors:** 


### Substitution.

There is possibly no man whose reputation can be more easily injured than a practicing physician's. The doctor's success depends entirely on his reputation for moral rectitude and professional knowledge, and for this reason he should be more than scrupulously careful in the administration of medicine. Medical men are possibly the only profession who place the custody of their implements and tools in the hands of others over whom they have practically no direction. The physician may diagnose ever so carefully, or prescribe ever so wisely, yet the moment that prescription leaves his hands the welfare of his patient is beyond his control. He may unite the skill of Osler, Pepper and Loomis in diagnosis, of Brunton, Wood and Hare in therapeutics, but it is all prostrate before the enlightened intelligence of some perfumed drug clerk. There are many noble leaders in pharmacy who have striven much to raise the drug trade to the dignity of a profession, and many of them have succeeded; but a chain is just as strong as its weakest link. The morale of a profession is at the mercy of its weakest member, and in the profession of pharmacy these are notoriously the substitutors. The substitutor is about the basest production of modern commerce; he has no respect for his own good name, his only interest is in his profits. To make a dime on a prescription he first defrauds the manufacturer who puts up a recognized and well-known article, robbing him of his good name by substituting inferior goods. He then robs the dying man because he does not give him that to which he is entitled. He steals from the physician his good name because he has given a worthless drug. The patient has not improved and the physician's reputation has suffered in consequence. The man who substitutes in any manner, shape or form can only be held in the eyes of the physician, the patient and the manufacturer as the meanest and most contemptible of sneak thieves.—Extract from editorial in *Dominion Medical Monthly* for February, 1897.

